# 

**Published:** 1898-07

**Authors:** 


### BUFFALO MEDICAL JOURNAL

Established 1845 by AUSTIN FLINT, M. D.

LIBRARY

SURGEON GENERAL'S OFFICE

JUL.-9--1898

EDITORS:

THOS. LOTHROP, M. D. WM. WARREN POTTER, M. D.

ASSOCIATE EDITORS:

WILLIAM C. KRAUSS, M. D.
JAMES WRIGHT PUTNAM, M. D.
JOHN A. MILLER, PH. D.
JOHN PARMENTER, M. D.
ERNEST WENDE, M. D.
ALVIN A. HUBBELL, M. D.

SCRIPTA FERUNT ANNOS

EUROPEAN AGENCY, J. B. BAILLIÉRE & FILS, 19 Rue Hautefeuille, Paris.
*Subscription, if paid in advance, $2.00; otherwise, $2.50. Foreign subscription, $2.50.*
Copyright 1898, by WILLIAM WARREN POTTER, M. D.
Entered at the Post Office at Buffalo, N. Y., as second-class mail matter.

A. T. BROWN PRINTING HOUSE, BUFFALO, N. Y.

Table of Contents on Page V.

